# Editorial: Fungal biology and related diseases

**DOI:** 10.3389/fmicb.2022.1037329

**Published:** 2022-10-21

**Authors:** Allan J. Guimarães, Lívia Kmetzsch, Marcos Dias Pereira

**Affiliations:** ^1^Department of Microbiology and Parasitology, Biomedical Institute, Fluminense Federal University, Niterói, Brazil; ^2^Rede Micologia RJ - Fundação de Amparo à Pesquisa do Estado do Rio de Janeiro (FAPERJ), Rio de Janeiro, Brazil; ^3^Bioscences Institute, Federal University of Rio Grande do Sul, Porto Alegre, Brazil; ^4^Biochemistry Department, Federal University of Rio de Janeiro, Rio de Janeiro, Brazil

**Keywords:** fungal biology, fungal metabolism, virulence, fungal gene expression, new antifungal strategies

The worldwide increasing incidence and mortality of fungal infections, including those by classical or (re-)emergent pathogens poses a serious public health problem. There is an urgent need for the implementation of compulsory notification of these neglected fungal diseases, besides a solid and strong investment on new efficient diagnostic and therapeutic alternatives to improve the control of mycoses. In this scenario, scientists have lately focused on decoding several aspects of fungal cells, including energy metabolism, cell cycle, gene expression, proteostasis, mitochondrial function, response to stress, secretory pathways and intrinsic or acquired resistance mechanisms. Therefore, for this Research Topic, we invited experts to share their latest findings on fungal biology: (i) metabolism and response to environmental changes; (ii) *in vivo* models of infection and virulence; (iii) gene expression and regulation; (iv) metagenomics and diagnosis and (v) strategies for the control of fungal infections.

As multifaceted organisms, fungi adapt to their environment, undergoing metabolic and morphological changes. Fabri et al. described pathways involved in the heat shock (HS) response linked to cell wall ultrastructural modifications and control of cell wall integrity (CWI) in *Aspergillus fumigatus*. In fact, expression of the HsfA and hsp90 is required for fungal adaptation to stress in *Aspergillus* biofilms. HsfA in turn, regulated the HS response gene expression and cell wall and lipid homeostasis, demonstrating its central role in the interplay of HS and the CWI cross-pathway regulation for *A. fumigatus* thermophily.

Adaptation to temperature was also explored by Almeida et al., on the thermally dimorphic fungus *Histoplasma capsulatum*. Comparative proteomics revealed that mycelia abundantly express glycolytic pathway and alcoholic fermentation enzymes, indicating the preferential utilization of anaerobic pathways for energy production, whereas yeast cells expressed tricarboxylic acid cycle and HS response proteins. Additionally, distinct expression of oxidative stress response or cell wall metabolism enzymes, differentially regulated the composition of this structure in the two morphologies of *H. capsulatum*.

Fungal metabolic adaptation, specifically lower funneling catabolism pathways of *Scedosporium* species, including the degradation of conserved aromatic intermediate compounds, were investigated by Poirier et al. The authors predicted several ring-cleaving dioxygenases in the *Scedosporium* genome, which were validated by the determination of expression levels of the gentisic acid cluster genes, overexpressed in the presence of lignin (or gentisic acid) and granting fungal environmental adaptation.

A key aspect of environmental adaptation and propagation of filamentous fungi is the proper spore formation. The mycopathogenic fungi *Trichoderma* produces chlamydospores, with enormous advantages over conidia regarding biotechnological applications. Peng et al. reported a time course transcriptomic analysis and differential gene expression during chlamydospore formation in *Trichoderma virens*, with emphasis on proper chitin synthesis for the cellular differentiation.

The characterization of fungal virulence and the host response to infection are necessary for the description of new therapeutics against fungi. Cryptococcal infection is dependent on the expression of several virulence determinants. Despite being well characterized, experimental murine cryptococcosis models do not mimic the human disease and relatively little is known when human infection is considered. de Sousa et al. reviewed the current procedures used to study Cryptococcus sp. virulence in humans, association and correlation of clinical patient data and characterization of fungal virulence and host-pathogen interactions, pointing out to the importance of international collaboration and data integration to overcome the difficulties and impulse the human cryptococcosis knowledge.

Considering fungal pathogens and plant models, species from *Fusarium* genera are known to infect a myriad of hosts, in which the associated diseases are hard to control. Damodaran et al. evaluate the application of the mycopathogen *Trichoderma reesei* to control *Fusarium* wilt in bananas, showing a high decrease in severity of the disease. This effect is possibly associated with increased production of antifungal compounds, and decreased production of *Fusarium* toxins.

Regarding the characterization of the expression of fungal virulence, agglutinin-like sequence (ALS) gene family of *Candida* species, including *Candida parapsilosis*, comprehends an important class of virulence determinants. However, due to the high sequence identity and the proportion of tandem repeats, correct assembly of these loci using high-throughput sequencing are generally unreachable. Oh et al. performed a mixed approach employing Oxford Nanopore MinION, Illumina MiSeq, and Sanger sequencing to fully uncover the ALS gene family in *C. parapsilosis*. Also, qRT-PCR assays were performed to evaluate expression of each ALS gene in *C. albicans, C. dubliniensis*, as well as *C. parapsilosis*, revealing a complex pattern of gene expression of the ALS gene family.

For the establishment of effective therapies to fungal infections, the correct identification of the etiological agent is necessary. Classical methodologies rely on culture and microscopical evaluations, which are time consuming and often non-specific and lack sensitivity. The description of new generation sequencing methodologies, including long-reads, at lower cost can accelerate the specific identification of pathogens in clinical samples. Hoang et al. discuss the pros and cons for the implementation of these techniques in the routine diagnosis of mycosis.

At last, understanding host responses and the proposal of new therapeutic strategies could help to control fungal infections more efficiently. Araújo et al. described how the administration of glucocorticoids impacted *C. neoformans* proliferation and its capsular structure, resulting in worst infections outcome in animal models, including higher organ fungal loads and shortened survival times, and could additionally explain the clinical failures in the setting of immunocompromised individuals. In the other hand, Granato et al. characterize the antifungal effects of 1,10-phenanthroline-5,6-dione (phendione) and its metal-based complexes against *Phialophora verrucosa*. While demonstrating additive effects to amphotericin B, their prominent effects on *P. verrucose* biofilms and their capacity to reduce infections to macrophages and fungal burden in *Galleria mellonella* models corroborate to their promising therapeutic potential.

In summary, the manuscripts in this collection cover many unexplored aspects of fungal biology being targeted for the development of new therapeutics and diagnosis, including new gene silencing and sequencing methodologies, omics and drug development approaches.

## Author contributions

All authors listed have made a substantial, direct, and intellectual contribution to the work and approved it for publication.

## Funding

AG was supported by FAPERJ (E-26/202.696/2018) and Conselho Nacional de Desenvolvimento Científico e Tecnológico (CNPq grant 309736/2021-8). LK was supported by Conselho Nacional de Desenvolvimento Científico e Tecnológico (CNPq. grant 312797/2021-4). MP was supported by Fundação Carlos Chagas Filho de Amparo à Pesquisa do Estado do Rio de Janeiro (FAPERJ, E-26/010001697/2019) and Conselho Nacional de Desenvolvimento Científico e Tecnológico (CNPq, grant 313783/2018-7).

## Conflict of interest

The authors declare that the research was conducted in the absence of any commercial or financial relationships that could be construed as a potential conflict of interest.

## Publisher's note

All claims expressed in this article are solely those of the authors and do not necessarily represent those of their affiliated organizations, or those of the publisher, the editors and the reviewers. Any product that may be evaluated in this article, or claim that may be made by its manufacturer, is not guaranteed or endorsed by the publisher.

